# First serological evidence of Q fever in large ruminants and its associated risk factors in Punjab, Pakistan

**DOI:** 10.1038/s41598-022-21405-y

**Published:** 2022-10-14

**Authors:** Sabir Hussain, Abrar Hussain, Muhammad Umair Aziz, Baolin Song, Jehan Zeb, F. M. Yasir Hasib, Jun Li, Abdul Rehman, David George, Alejandro Cabezas-Cruz, Olivier Sparagano

**Affiliations:** 1 Department of Infectious Diseases and Public Health, Jockey Club College of Veterinary Medicine and Life Sciences, City University of Hong Kong, Kowloon, Hong Kong SAR China; 2 Department of Pathobiology, College of Veterinary Medicine, University of Illinois, Urbana-Champaign, 61802 USA; 3 Department of Epidemiology and Public Health, University of Veterinary and Animal Sciences, Lahore, 54600 Pakistan; 4 School of Natural and Environmental Sciences, Newcastle University, Newcastle upon Tyne, NE1 7RU UK; 5 Laboratoire de Santé Animale, Anses, INRAE, Ecole Nationale Vétérinaire d’Alfort, UMR BIPAR, 94700 Maisons-Alfort, France

**Keywords:** Microbiology, Zoology, Diseases, Medical research, Risk factors

## Abstract

Coxiellosis, also known as Q fever, is a zoonotic disease caused by *Coxiella burnetii*, a gram-negative bacterium that exerts a significant deleterious impact on the productive and reproductive capabilities of livestock, severely effecting the economics of this sector. In this study, 448 sera samples from cattle (n = 224) and buffalo (n = 224) were collected from 112 farms in Pakistan and examined for antibodies against *C. burnetii* using an indirect ELISA. Ticks were also collected from these animals. Serological analysis revealed a 23.66% and 27.23% seroprevalence of Q fever in cattle and buffalo, respectively. Odds ratio (OR) analysis of the factors associated with *C. burnetii* seropositivity was performed, and a multivariable logistic model identified five main variables associated with the seropositivity for coxiellosis. These were: (i) the absence of acaricide use (OR 5.61; 95% CI 2.97–10.94); (ii) the presence of ticks (OR 3.23; 95% CI 1.87–5.69); (iii) the abortion history during the preceding year on the farm (OR 14.96; 95% CI 8.09–29.34); (iv) the presence of sheep and goats (OR 2.47; 95% CI 1.20–5.35); and (v) the absence of a separate parturition area (OR 3.17; 95% CI 1.76–5.86). This study provides new insights into the seroprevalence of Q fever in large ruminants across seven studied districts of Punjab, Pakistan, also providing baseline data to inform improved herd management and on-farm practices for the prevention and control of Q fever in large ruminants in the region. Results of this work suggest that further molecular investigation of coxiellosis is warranted to provide a more thorough evaluation of *C. burnetii* epidemiology in Pakistan.

## Introduction

Coxiellosis, also known as Q fever, is a worldwide zoonotic disease caused by *Coxiella burnetii*, an intracellular gram-negative bacterium categorized as a biological (Type B) warfare agent^1^. Coxiellosis is known to occur throughout the globe, with the only countries not reporting cases to date being New Zealand, French Polynesia, Ireland, the Netherlands, Poland, and the Scandinavian countries^2–4^. Several epidemiological studies have increased public health awareness of Q fever^5, 6^, with cases reported in humans worldwide, including 14 in Switzerland^7^ and 10 (in military personnel) in France^8^. A staggering 4,000 acute and 284 chronic human Q fever cases have been reported in the Netherlands, with cases reaching epidemic proportions as a probable result of both seropositive blood donors and affected populations living in close proximity to ovine herds^9^. Some domestic animals, mainly sheep and goats, can act as reservoirs for Q fever^10^, with asymptomatic infection suggested in ruminants based on reports of serologically negative dairy cows^11^. In large ruminants, coxiellosis causes premature birth, sporadic abortions, dead or weak calf, plancentitis, and subclinical mastitis, causing significant economic losses in herds^12^. The signs and symptoms observed in animals per se include abortion, infertility, stillbirth, mastitis, metritis, weak-offspring, and induced reproductive disorder in domestic ruminants, culminating again in economic impacts^13–17^. Where Q fever causes ovine and caprine abortions in herds^18, 19^, *C. burnetii* are shed via birth products (e.g. birth fluids and the placenta). This creates a source of contamination that increases the risk of related outbreaks in humans^19, 20^, though the bacterium may also be shed through ruminant urine^21^, milk products and vaginal mucus and feces^22^. Ticks also play a role in the transmission of *C. burnetii* in animals, but their role as vectors of this bacterium in humans is not fully understood^23^.

Q fever is highly contagious for humans, especially those working in close contact with infected ruminants, such as abattoir staff, veterinarians, and farmers^24, 25^. In humans, Q fever can lead to an acute self-limiting disease with flu-like symptoms, or to a chronic disease associated with hepatitis, endocarditis (in immuno-compromised patients), encephalitis, abortions and stillbirth in pregnant women^26^. *Coxiella*-infected cows may also develop metritis, infertility, and mastitis, and may have the potential to shed *C. burnetii* through milk for up to 32 months^27^. Infected animals are thought to transmit Q fever to humans via an aerosol route^28^, though some, albeit negligible, oral transmission has been reported (often via consumption of contaminated dairy products)^29^, with sexual and vertical transmission also being possible once an individual is infected^30, 31^.

Serological diagnosis of Q fever is achieved using immunofluorescence assays (IFA), enzyme linked immunosorbent assays (ELISA) or complement fixation tests (CFT)^32^. Direct detection of *C. burnetii* can be achieved by polymerase chain reaction (PCR) or in vitro culture of the bacterium. Isolation of the Q fever pathogen is a reliable diagnostic method, but is difficult, time consuming and hazardous, also requiring access to BSL 3 facilities^26^. According to the World Animal Health Organization (OIE), CFT and ELISA have better sensitivity and specificity than IFA for Q fever detection^33^. The CFT is, however, time consuming, tedious and requires specific laboratory conditions, while ready-to-use ELISA kits are widely available and the preferred diagnostic test according to recommendations from the OIE (made in 2015). In veterinary medicine, indirect diagnostic methods such as ELISA are commonly used to detect antibodies against *C. burnetii*, and for that purpose different kits are available with different sensitivity and specificity ranges^34–44^. Such kits are not without potential flaws, however, and it must be borne in mind that cross-reactions with other pathogens, such as *Bartonella quintana*, *Bartonella henselae*, and *Legionella micdadei*, can influence test specificity^44–48^.

In Pakistan, the agricultural sector (including crops and livestock) employs 45% of the national workforce and produces 21% of the national Gross Domestic Production (GDP)^49^. Livestock farming constitutes 56% of the agriculture sector and 12% of the GDP, playing an important role in poverty alleviation and economic growth^49^. In rural areas, the majority of the population relies on livestock for their livelihood^50^, with Pakistan being the 4th largest milk-producing country in the world, and milk being known nationally as ‘White Gold’^50^. Almost 48 million cattle and 40 million buffalo are owned by rural families or smallholder farmers in Pakistan^51^, though the first case of Q fever in the country, in 1955, was reported in camels^12^. Across Pakistan, other diseases such as brucellosis also cause reproductive disorders in livestock^52–54^, making the diagnosis and differentiation of *Coxiella* challenging.

Coxiellosis is considered a neglected disease in both humans and animals in Pakistan. The majority of coxiellosis cases in the country are overlooked, due to either the lack of a proper diagnosis or misdiagnosis for other diseases with similar symptoms, for example brucellosis that can present with fever and abortion^1^. From 1955 to 2019, only two studies of coxiellosis (Q fever) were undertaken in large ruminants of Pakistan, and in both cases antigen was detected using CFT and ELISA^55, 56^. According to the limited available literature, the seroprevalence of Q fever in Pakistan ranges from 4.6 to 40% in all livestock species, and from 10.2 to 26.8% in humans^55, 56^. The rates of premature births and weak calves are particularly high in certain districts, including those selected for study in the current work^57^ which was designed to fully assess Q fever seroprevalence and associated factors in large ruminants on livestock farms in Punjab, Pakistan. In estimating the seroprevalence of coxiellosis in seven districts not previously targeted in preceding research, this study provides essential information for policy makers and concerned authorities to implement prevention and control strategies against coxiellosis in Pakistan.

## Materials and methods

### Ethical approval

This study obtained ethical approval from the Institutional Research Ethics Committee of the City University of Hong Kong with internal reference number A-0672. All procedures were performed in accordance with the relevant guidelines and regulations. This study was conducted in accordance with (ARRIVE) Animal Research: Reporting In Vivo Experiments.

### Study population

Pakistan has five provinces with Punjab being the largest, containing 36 districts and the highest animal and human populations in the country. This study was conducted across various smallholder livestock farms from October 2020 to January 2021, surveying sites where large ruminants were present (n = 112) across the following seven districts of Punjab: Khushab, Vehari, Sheikhupura, Muzaffargarh, Kasur, Gujranwala and Bahawalnagar. Livestock farms and animals residing on them were selected randomly using the software Survey Toolbox (Ausvet, The Australian Biosecurity Cooperative Research Centre for Emerging Infectious Disease, Australia)^58^. Farms were chosen on the basis of operational convenience in all districts, where selected farms-maintained herds of between 5 and 60 animals. From 112 farms (16 from each district), 448 animals were sampled; 224 cattle and 224 buffaloes. Geographically, this province covers an area of 205,344 km^2^ and is located 31.1704° N and 72.7097° E in a semiarid lowland region. The average temperature ranges from a minimum of − 2 °C to a maximum of 46 °C, but can reach − 10 °C in winter and 50–52 °C in summer^1^. Livestock is the primary source of income in rural areas of Pakistan and, as previously noted, is a major contributor to the country’s economy. Punjab itself is a key production region for many species, being home to 24% of Pakistan’s sheep, 37% of its goats, 49% of its buffalo and 65% of the country’s cattle.

### Sample size calculation

Coxiellosis seroprevalence in large ruminants in the project study areas was unknown prior to commencing work. As such, sample size was calculated using 95% confidence intervals (CI), with an expected seroprevalence of 50% and an absolute precision of 5%, as recommended by Thursfield^59^.

$${\text{N }} = \, \left( {{1}.{96}} \right)^{{2}} {\text{P }}\left( {{1 } - {\text{ P}}} \right)/{\text{L2}}.$$

N indicates sample size, where 1.96 is the Z value for the selected CI (95%), P is the expected disease seroprevalence and L is the desired absolute precision. Using this approach, from all livestock farms, 384 serum samples were calculated as being required. A total of 112 farms from seven districts were visited, where for each district, sixteen farms were selected randomly, and on each four animals (two cattle and two buffalo) were randomly sampled. In this way, 448 serum samples were collected from study area (224 from cattle and 224 from buffalo). During collection of samples, data was also obtained from the farm owner using a pre-designed questionnaire.

### Sample collection

For blood collection, four blood samples were collected per farm from four animals (two from cows, two from buffaloes), with preference given to sampling those animals which were infected with ticks. Approximately 8 to 10 ml of blood was collected from the jugular vein of each animal using disposable needles. Blood was collected in thrombin-containing vacutainers (Catalog No. VP20021S, BD Diagnostic, Oxford, UK) to easily separate serum from red blood cells. Samples were carefully labeled according to each district, farm, and animal. Blood samples were then stored at 4 °C and transported to the University of Veterinary and Animal Sciences, Lahore, Pakistan. The samples were centrifuged at 3000 rpm for 5–10 min for serum separation, with serum then extracted and added into 1.5 ml Eppendorf safe-lock tubes (Catalog No. 0030123328, Sigma-Aldrich, Missouri, United States). Sera samples, conserved at − 20 °C, were then shipped to the Department of Infectious Diseases and Public Health, City University of Hong Kong.

### Tick collection

Alongside blood sample collection, 358 ticks (182 from 92 cows and 176 from 87 buffaloes) were collected from the same animals in the seven districts of Punjab, Pakistan. Tick collection was done during the winter season (from October to January) when tick infestation is rare and, consequently, low numbers of ticks were typically present on infested animals, dictating that two ticks per animal were collected to maintain uniformity. The ticks from each animal were placed into labeled Eppendorf tubes (3 ml) containing 70% ethyl alcohol as a preservative, prepared at the University of Veterinary and Animal Sciences, Lahore, Pakistan. Ticks were then shipped to City University of Hong Kong, following international regulations for transportation and after acquisition of a Hong Kong Department of Health import permit, where specimens were identified to species level under a stereomicroscope. Two complementary identification keys where used, these being a hard copy of Walker et al.^60^ and an online taxonomic key included in Multikey 2.1^61, 62^, with reference also made to original descriptions and re-descriptions of relevant tick species.

### Questionnaire data collection

Survey data was collected from each farm using a predesigned questionnaire, with this data subsequently used for odds ratio analysis. Each question was translated into the local language to avoid confusion and maximize accuracy and pretested on selected farmers before circulation. The questionnaire gathered metadata on the district and farm name, the total number of animals kept at the farm, common practices such as feeding methods employed (stallfed, grazed, mixed), acaricidal use and frequency of using these acaracides, abortion history, how many abortions occurred in the preceding year, presence of sheep and goats on the farm, and separate parturition area presence/absence. Details on the age, parity and reproductive status of the sampled animals were also recorded.

### Serological test

ID Screen Q Fever Indirect Multi-species (ID vet, France) was used for the detection of antibodies of Q fever in all serum samples, as per manufacturer recommendations. The plates were read at 450 nm with an ELISA reader (SpectraMax iD3, San Jose, California, USA), also as per manufacturer recommendations, with these readngs automatically downloaded in an Excel file connected to the machine. The test was considered to be valid if: (i) the mean positive control optical density (OD_PC_) was greater than 0.350, and (ii) the ratio of the mean value of the positive control (PC) OD to the negative control OD (OD_PC_ to OD_NC_) was greater than 3. Results were interpreted as detailed in Table 1.

**Table 1:** Result interpretation of ELISA for Q fever.

S/P% values*	Interpretation of result
S/P% ≤ 40	Negative
40 < S/P% ≤ 50	Doubtful
50 < S/P% ≤ 80	Positive
S/P% 80 > 80	Strong positive

*The sample (S) to positive (P) was estimated using the following formula: S/P = ((OD _Sample_ − OD_NC_)/(OD_PC_ − OD_NC_) × 100).

Only ‘positive’ and ‘strong positive’ (Table 1) results were included for the calculation of seroprevalence.

### Statistical analysis

From the seven selected districts of Punjab, 112 farms were sampled with herd sizes ranging between five and 60 animals. Descriptive statistical analysis was applied to determine the seroprevalence of herds for antibodies again *C. burnetii*. A herd was considered positive when a single animal was positive to the ELISA test.

All data collected through predesigned questionnaires was entered in an Excel file (Microsoft Excel 2016), which was then imported into open-source R software (version 3.2.3). Univariate analysis was conducted to study the association between coxiellosis seropositivity and thirteen independent variables (Table 4) included for odds ratio analysis. Of these thirteen variables, only seven variables were selected for initial multivariate analysis, using the selection criteria of *p* < 0.2 to check their contribution towards seropositivity. In the initial multivariate model, all variables with *p* > 0.05 were excluded sequentially and their effect on odds ratios and *p-*values of other predictors were noted. Finally, a multivariate model was developed with five variables that had proven to be significant predictors of Q fever seropositivity at *p* < 0.05.

## Results

### Seroprevalence of coxiellosis

**Figure 1:**
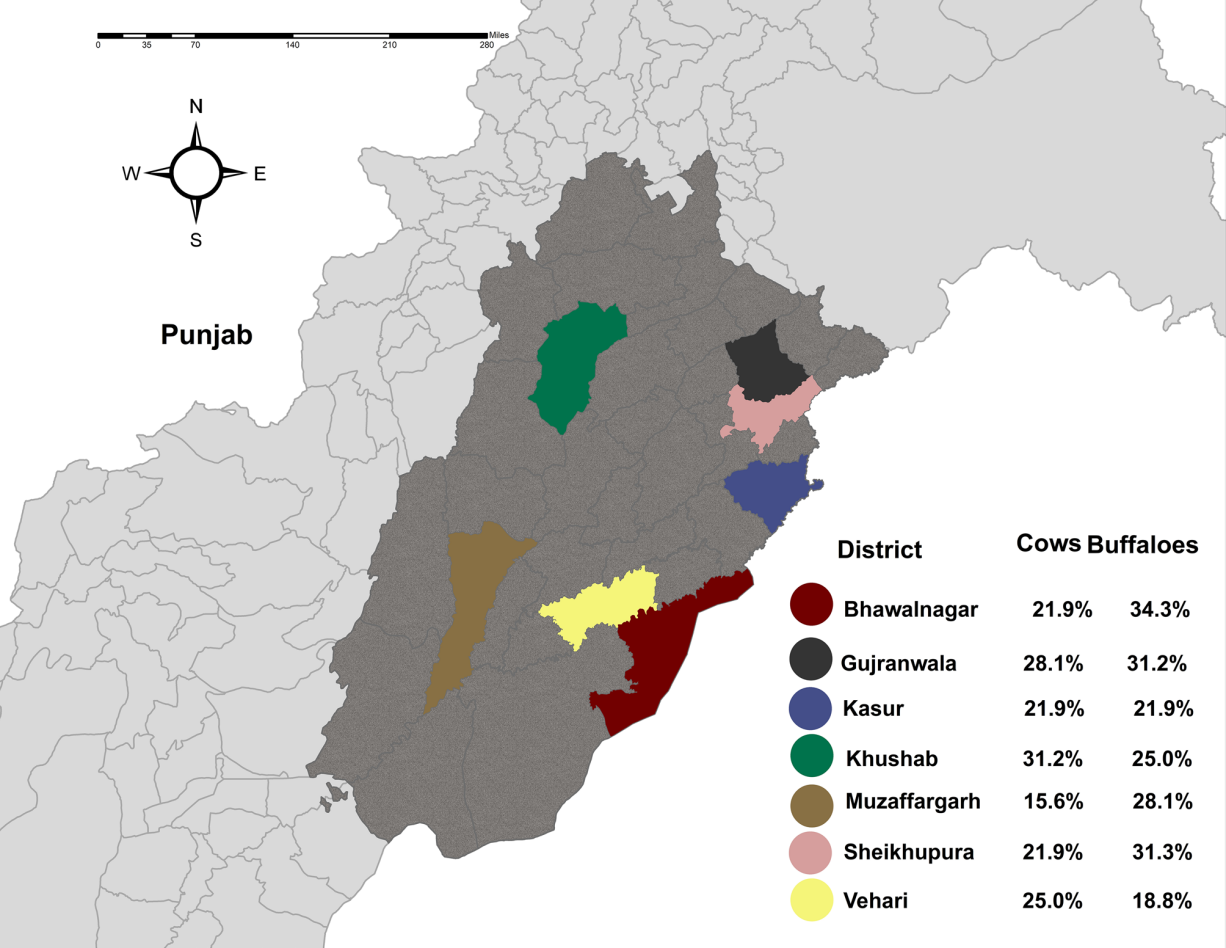
Seroprevalence of Q fever in large ruminants in seven districts of Punjab, Pakistan.

From the seven studied districts of Punjab, Pakistan, the farm level seroprevalence of coxiellosis (i.e. the percentage of farms with at least one positive case) was recorded as being 58.92% (66/112, 95% CI 49.22–68.01), with animal-based seroprevalence (i.e. the percentage of seropositive animals) being 25.44% (114/448, 95% CI 21.52–29.79). Coxiellosis in buffaloes was numerically higher at 27.23% (61/224, 95% CI 21.61–33.64) than in cattle at 23.66% (53/224, 95% CI 18.36–29.87), with the numerically highest prevalence (29.68%, 10/32, 95% CI 19.24–42.58) recorded in the district of Gujranwala, and the lowest prevalence (21.87%, 7/32, 95% CI 12.8–34.28) seen in the districts of Kasur, Muzaffargarh and Vehari. At the farm level, the numerically highest seroprevalence (81.25%, 13/16, 95% CI 53.69–95.02) was recorded in the district of Khushab and the lowest seroprevalence (31.25%, 5/16, 95% CI 12.13–58.51) in the district of Vehari (Fig. 1) (Tables 2, 3).

**Table 2:** Animal level seroprevalence of Q fever in large ruminants in Punjab, Pakistan.

Districts	Positive/tested (cow)	Positive/tested (Buffalo)	Total positive/tested	Prevalence (%)	95% CI
Kasur	7/32	7/32	14/64	21.87	12.80–34.28
Sheikhupura	7/32	10/32	17/64	26.56	16.65–39.31
Gujranwala	9/32	10/32	19/64	29.68	19.24–42.58
Muzaffargarh	5/32	9/32	14/64	21.87	12.80–34.28
Vehari	8/32	6/32	14/64	21.87	12.80–34.28
Khushab	10/32	8/32	18/64	28.12	17.92–40.95
Bahawalnagar	7/32	11/32	18/64	28.12	17.92–40.95
Total	53/224	61/224	114/448	25.45	21.52–29.79

**Table 3:** Farm level seroprevalence of Q fever in ruminant farms in Punjab, Pakistan.

Districts	Positive/tested	Prevalence (%)	95% CI	*p*-value
Kasur	9/16	56.25	30.55–79.24	0.80
Sheikhupura	11/16	68.75	41.48–87.88	0.21
Gujranwala	10/16	62.50	35.87–83.71	0.45
Muzaffargarh	8/16	50.00	27.99–72.00	1.00
Vehari	5/16	31.25	12.13–58.51	0.21
Khushab	13/16	81.25	53.69–95.02	0.024
Bahawalnagar	10/16	62.50	35.87–83.71	0.45
Total	66/112	58.92	49.22–68.01	0.11

### Odds ratio analysis

The univariate analysis conducted indicated significant associations between seropositivity of Q fever with no acaricide use (OR 4.79, CI 95% 2.91–7.93, *p* < 0.001) and tick presence (OR 3.28, CI 95% 2.12–5.13, *p* < 0.001). The presence of sheep and goats (OR 2.36, CI 95% 1.37–4.29, *p* = 0.002), abortion history from the preceding year at the farm (OR 8.87, CI 95% 5.39–15.10, *p* < 0.001), and absence of a separate parturition area (OR 1.83, CI 95% 1.16–2.96, *p* = 0.010) were also significantly associated with seropositivity of Q fever. Other factors, including keeping a mixed herd (OR 0.82, CI 95% 0.53–1.96, *p* = 0.389), milk reduction during tick presence (OR 0.85, CI 95% 0.53–1.40, *p* = 0.531), presence of a quarantine facility (OR 0.886 CI 95%: 0.56–1.39, *p* = 0.595) and sandy floor (OR 2.54, CI 95% 1.60–3.90, *p* = 2.540), vaccination practices (for Foot and Mouth Disease (FMD), hemorrhagic septicemia (HS), black quarter (BQ), brucellosis, theileriosis, and anthrax, as recommended by the Livestock Department of Punjab, Pakistan) (OR 0.77, CI 95% 0.38–1.64, *p* = 0.489), presence of a breeding bull (OR 1.58, CI 95% 0.91–2.71, *p* = 0.095) and reproductive status (OR 1.47, CI 95% 0.95–2.27, *p* = 0.080) were not significantly associated with Q fever seropositivity in univariate analysis (Table 4).

**Table 4:** Summary of the variables included in the univariable analysis to test for association with ELISA positive Q fever results.

Variables	Categories	Positive/tested	Prevalence % (95% CI)	Odds ratio	95% CI	*p-*value
Keeping animals together	Yes	51/216	23.61 (18.23–29.95)	0.82	0.53–1.96	0.389
	No	63/232	27.15 (21.64–33.44)	Ref		
Acaricide use	Yes	69/363	19.00 (15.17–23.50)	Ref	2.91–7.93	< 0.001*
	No	45/85	52.94 (41.86–63.74)	4.79		
Tick presence	Yes	70/179	39.10 (31.99–46.69)	3.28	2.12–5.13	< 0.001*
	No	44/269	16.35 (12.25–21.44)	Ref		
Milk reduction during tick presence	Yes	83/336	24.70 (20.25–29.73)	0.85	0.53–1.40	0.531
	No	31/112	27.67 (19.84–37.06)	Ref		
Abortion occurred last year on the farm	Yes	91/194	47.64 (39.76–54.17)	8.87	5.39–15.10	< 0.001*
	No	23/254	9.05 (5.94–13.44)	Ref		
Quarantine facility	Yes	41/152	26.97 (20.25–34.87)	Ref	0.56–1.39	0.595
	No	73/296	24.66 (19.94–30.05)	0.886		
Type of floor	Sandy	61/165	36.96 (29.69–44.86)	2.54	1.60–3.90	2.540
	Concreted	53/283	18.72 (14.45–23.87)	Ref		
Presence of sheep and goats	Yes	97/333	29.12 (24.36–34.38)	2.36	1.37–4.29	0.002*
	No	17/115	14.78 (9.09–22.89)	Ref		
Presence of breeding bull	Yes	24/72	33.33 (22.92–45.53)	1.58	0.91–2.71	0.095*
	No	90/376	23.93 (19.77–28.63)	Ref		
General vaccination practices	Yes	12/40	30.00 (17.08–46.71)	Ref	0.38–1.64	0.489
	No	102/408	25.00 (20.93–29.54)	0.77		
Separate parturition area	Yes	31/167	18.56 (13.13–25.47)	Ref	1.16–2.96	0.010*
	No	83/281	29.53 (24.34–35.30)	1.83		
Animal tested	Cow	53/224	23.66 (18.36–29.87)	Ref	0.54–1.27	0.386
	Buffalo	61/224	27.23 (21.61–33.64)	0.83		
Reproductive status of animal	Pregnant	49/162	30.24 (23.41–38.03)	1.47	0.95–2.27	0.080*
	Non-pregnant	65/286	22.72 (18.09–28.11)	Ref		

*Ref* reference category.

Significant values are in bold.

All seven of the variables having *p-*value less than 0.2 in univariate analysis were included in an initial multivariate model, where all variables with *p* > 0.05 were then excluded sequentially and their effect on odds ratios and *p-*values of other predictors noted. In the first step, for example, ‘presence of breeding bull’ was excluded, and the effect of this action on the odds ratio and *p-*values of the other factors noted, with this approach then repeated to next exclude ‘reproductive status of the animal’. The end result of this process was a final model containing five predictors that had proven to be significant predictors of Q fever seropositivity at *p* < 0.05, these being; no acaricide use (OR 5.61; 95% CI 2.97–10.94), presence of ticks (OR 3.23; 95% CI 1.87–5.69), abortion history in the preceding year at the farm (OR 14.96; 95% CI 8.09–29.34), presence of sheep and goats (OR 2.47; 95% CI 1.20–5.35), and absence of a separate parturition area (OR 3.17; 95% CI 1.76–5.86) (Table 5).

**Table 5:** Final multivariate model including significant predictors of Q fever seropositivity.

Predictors	Odds ratio	95% CI	*p*-value
Acaricide use (no)	5.61	2.97–10.94	< 0.001
Tick presence (yes)	3.23	1.87–5.69	< 0.001
Abortion in the preceding year (yes)	14.96	8.09–29.34	< 0.001
Presence of sheep and goats (yes)	2.47	1.20–5.35	0.017
Separate parturition area (no)	3.17	1.76–5.86	< 0.001

### Tick identification

**Figure 2:**
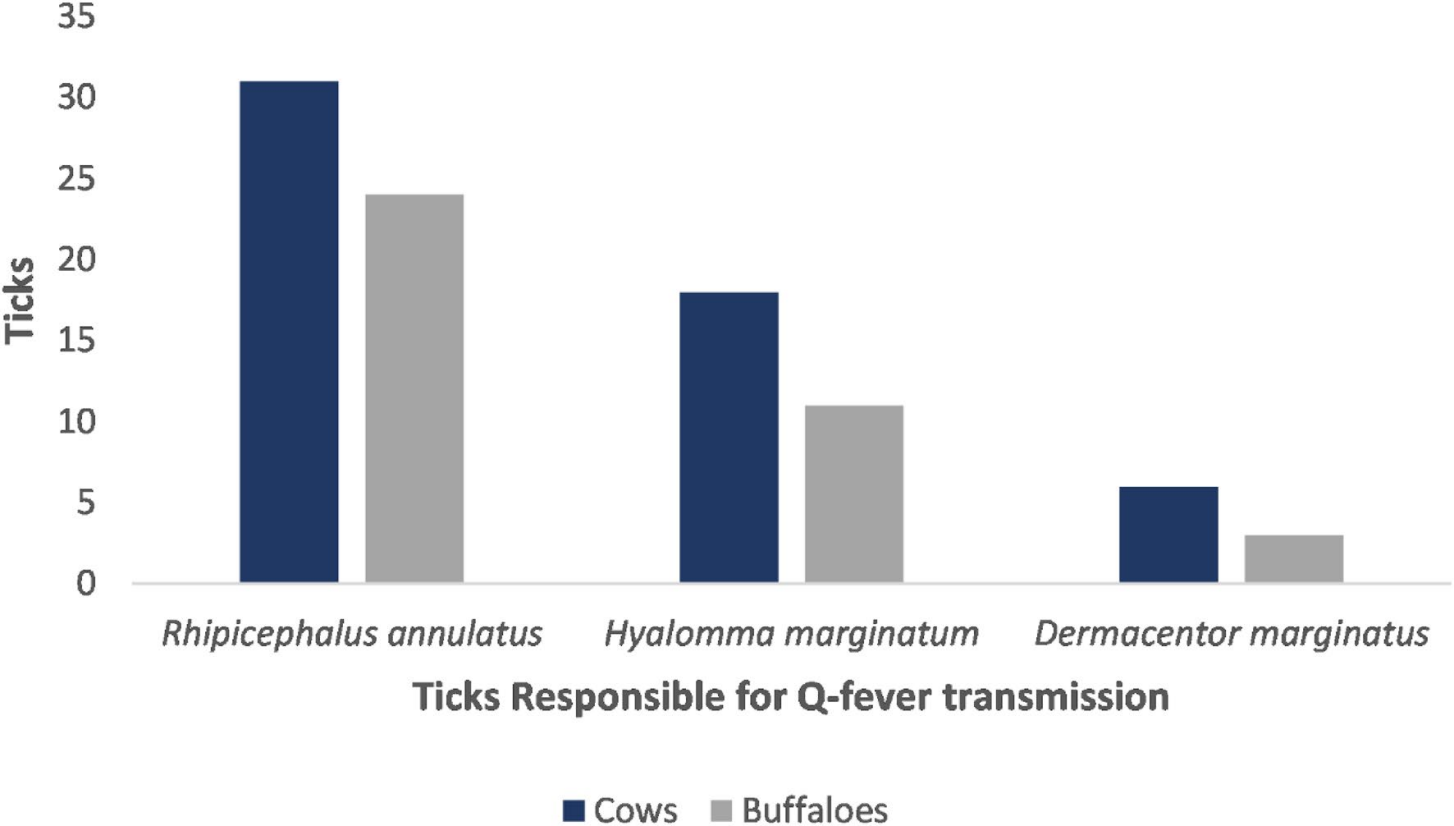
Ticks responsible for Q fever.

All tick samples were morphologically identified. Almost 20.7% (93/448) of animals included in the study were infested with one or more of *Rhipicephalus annulatus, Dermacentor marginatus* and *Hyalomma marginatum*, all of which have been reported as major vector of Q fever, and the presence of which supports that these ticks might carry this pathogen and be responsible for its transmission (Fig. 2). Almost 19.3% (86/448) of animals were infected with other tick species (*Hyalomma scupense,* and *Hyalomma truncatum*) which are not known to be responsible for Q fever transmission, but which may be associated with other tick-borne diseases (Table 6). Conversely, 60.0% (269/448) of animals were not infected with ticks at the time of sampling. A Chi-square test found a significant association (χ^2^ = 61.95, *p* < 0.001) between the presence of tick species previously reported as responsible for Q fever transmission (i.e. *Rhipicephalus annulatus, Dermacentor marginatus* and *Hyalomma marginatum*) and ELISA positive results for Q fever in animals (Table 7).

**Table 6:** Identified tick species from cattle and buffaloes.

Identified tick species	Cows	Buffaloes	Responsible for Q fever transmission	References from already available studies
*Rhipicephalus annulatus*	31	24	Yes	^63, 64^
*Hyalomma marginatum*	18	11	Yes	^63–65^
*Dermacentor marginatus*	6	3	Yes	^66–70^
*Hyalomma scupense*	19	9	No	^71^
*Hyalomma truncatum*	37	21	No	^71^

**Table 7:** Association of tick species with seroprevalence of Q fever with χ^2^ test.

Variable	Positive	Negative	Odds ratio (95% CI)	*p*-value
Animals with ticks responsible for Q fever transmission	53	40	5.38 (4.65–6.43)	< 0.001
Animals with tick are not responsible for Q fever transmission	17	69	0.19 (0.11–0.24)	
Animals with no ticks	44	225	0.15 (0.9–0.18)	

## Discussion

This is the first study to estimate the seroprevalence of Q fever in cattle and buffaloes in the selected seven districts of Punjab, Pakistan, revealing an overall seroprevalence of 25.45% (95% CI: 21.52–29.79) in the bovine population. In cattle, seroprevalence was lower (23.66%) than in buffaloes (27.23%), in agreement with earlier studies in Punjab, India, where respective seroprevalences of 23.2% and 24.1% were recorded^72^. On a global scale a slightly lower seroprevalence in cattle has been reported (20%)^12^, though with figures varying widely between countries and being higher than recorded in the current work in many cases (24% in Canada, 39% in the Netherlands, 40% in Germany, 46% in Japan and 82% in the USA)^73–76^. Lower seroprevalence among Pakistan’s cattle and buffaloes is suggested in a 2019 study by Rashid et al., with only 6.1% of animals testing positive for Q fever across 11 dairy farms^77^. This notably low figure probably resulted from this work only encompassing institutional holdings (managed by government authorities), where animal management would have been delivered by qualified veterinarians. This is in contrast to the current study, which surveyed non-institutional, commercial operations and small-holder farms to provide a better indication of seroprevalence across the sector as a whole. Nevertheless, that this variation in seroprevalence might also be due to sampling different geographical areas under varying environmental conditions^78, 79^ cannot be discounted. Variation in Q fever seroprevalence between bovines and additional animal species in Pakistan could also be expected based on the research elsewhere. Hussain et al., for example, detected anti-C. burnetii antibodies in 288 of 920 camels sampled in Pakistan (31.3%, 95% CI 28.3–34.4%)^57^, suggesting higher seroprevalence in this species than in the cattle/buffalo population sampled in the current study (25.45%, 95% CI 21.52–29.79). Work has also been done in this region to suggest that C. burnetii is widespread outside of its living hosts, being detectable by DNA analysis in 47 of 2,425 soil samples taken by Shabbir et al.^1^, though with soils testing positive at a notably lower rate than animals (i.e. 1.94%, 95% CI ± 0.55, versus the higher figures noted above for cattle, buffaloes and camels).

Ticks are considered major reservoirs of *C. burnetii* and are responsible for the transmission of coxiellosis to domestic and wild animals^80, 81^*.* The multiplication of *C. burnetii* in the mid-gut of infected ticks has been demonstrated, with the bacteria being present throughout the entire life of the tick and transovarial transmission to the next generation progeny being a possibility^82^. The findings of the current study support an association between ticks and coxiellosis, with the latter being three times more likely to occur where the former is found. Moreover, farms that did not implement the use of acaricides were five times more likely to be positive for coxiellosis according to our final multivariate model.

Various studies from numerous countries around the globe have found ticks to be positive for coxiellosis^56, 83–86^, with Duron et al. reporting that over 40 tick species can serve as vectors of Q fever^81^. In Pakistan, coxiellosis has been reported in tick pools collected from sheep and goats with seroprevalences of 31.0% and 7.7%, respectively^23^. We found that *Rhipicephalus annulatus, Dermacentor marginatus* and *Hyalomma marginatum* were the major tick species found on those farms that were seropositive for coxiellosis, with work elsewhere using molecular techniques to confirm that these species can vector coxiellosis^63, 68^, this being consistent with our results. According to Browne et al., acaricide use reduces tick population feeding on cattle^87^, which is also in accordance with our finding that a lack of acaricide use on the farm can increase the chances of Q fever.

The current study reported that a history of on-farm abortions in livestock poses 14× greater odds of seropositivity for Q fever. Similar results have been reported elsewhere, and can be explained by the link to reproductive disorders often seen with Q fever; for example, seropositivity of *C. burnetii* has been reported as significantly associated with reproductive disorders in livestock from France^88^, the Netherlands^73^, Hungary^89^, Germany^90^, and Japan^91^. According to a study conducted in Italy, the distribution of seropositivity in cows and abortion of calves was linked, with peak abortions observed in seasons of higher tick prevalence in the study area^92^.

The current study also demonstrated, in the final multivariate model, that farms having separate parturition areas were three times less likely to have coxiellosis. Another study conducted in Egypt reported that presence of abortion and parturition material can contribute significantly to transmission of Q fever^93^. Reproductive disorders, abortion, and parturition material should therefore be managed carefully to reduce the odds of coxiellosis spread, and to this end a separate parturition area on the farm can be recommended.

Presence of sheep and goats also presents a risk of coxiellosis according to the current study, where these animals can also harbor *C. burnetii*. A study conducted in Iran demonstrated a relatively high seroprevalence of Q fever in small ruminants with a history of abortion^94^. Nevertheless, the seroprevalence of coxiellosis in ovines can vary significantly between countries, with past research suggesting figures of 20% in France, 3.5% in the Netherlands, 8.7% in Germany and 56.9% in Bulgaria^95^. Caprine seroprevalence may similarly vary, having been reported as 7.8% in the Netherlands, 2.5% in Germany, and 40% in Bulgaria^95^. Such studies confirm that Q fever is commonly prevalent in small ruminants. In the case of Pakistan, recent research has reported 15.6% and 15% seroprevalence in sheep and goats respectively^56^, explaining links between the presence of these animals and the occurrence of coxiellosis in larger livestock in the current study.

## Conclusions

This is the first study to explore the seroprevalence and associated risk factors of coxiellosis in large ruminants in the seven selected districts sampled in Punjab, Pakistan. This work provides baseline data and valuable insight into the major contributing factors that drive seropositivity of coxiellosis in large ruminants in this region. Based on these findings it can be recommended that abortions in herds should not be neglected, with proper screening undertaken to evaluate the cause. This, and other measures, should reduce the burden of coxiellosis, and other livestock diseases similarly associated with reproductive disorders. Tick management, achieved through acaricide use or other means, can also play a vital role in management, as can implementing hygienic measures that can reduce Q fever contamination and spread, and minimize movement of *C. burnetii* from small to large ruminants.

## Supplementary Information

Supplementary Information.

## Data Availability

All data generated or analysed during this study are included in this published article (and its Supplementary Information files).
